# CRIS—A Novel cAMP-Binding Protein Controlling Spermiogenesis and the Development of Flagellar Bending

**DOI:** 10.1371/journal.pgen.1003960

**Published:** 2013-12-05

**Authors:** Anke Miriam Krähling, Luis Alvarez, Katharina Debowski, Qui Van, Monika Gunkel, Stephan Irsen, Ashraf Al-Amoudi, Timo Strünker, Elisabeth Kremmer, Eberhard Krause, Ingo Voigt, Simone Wörtge, Ari Waisman, Ingo Weyand, Reinhard Seifert, Ulrich Benjamin Kaupp, Dagmar Wachten

**Affiliations:** 1 Center of Advanced European Studies and Research (caesar), Department of Molecular Sensory Systems, Bonn, Germany; 2 University of Göttingen, Third Institute of Physics - Biophysics, Göttingen, Germany; 3 German Center for Neurodegenerative Diseases (DZNE), Bonn, Germany; 4 Helmholtz Zentrum München, German Research Center for Environmental Health, Institute of Molecular Immunology (IMI), Munich, Germany; 5 Leibniz-Institut für Molekulare Pharmakologie (FMP), Berlin, Germany; 6 Max Planck Institute for Biology of Ageing, Transgenic Core Facility, Cologne, Germany; 7 Institute for Molecular Medicine, University Medical Center of the Johannes Gutenberg-University of Mainz, Mainz, Germany; 8 Institute of Complex Systems, Cellular Biophysics (ICS-4), Forschungszentrum Jülich, Jülich, Germany; University of Washington, United States of America

## Abstract

The second messengers cAMP and cGMP activate their target proteins by binding to a conserved cyclic nucleotide-binding domain (CNBD). Here, we identify and characterize an entirely novel CNBD-containing protein called CRIS (cyclic nucleotide receptor involved in sperm function) that is unrelated to any of the other members of this protein family. CRIS is exclusively expressed in sperm precursor cells. *Cris*-deficient male mice are either infertile due to a lack of sperm resulting from spermatogenic arrest, or subfertile due to impaired sperm motility. The motility defect is caused by altered Ca^2+^ regulation of flagellar beat asymmetry, leading to a beating pattern that is reminiscent of sperm hyperactivation. Our results suggest that CRIS interacts during spermiogenesis with Ca^2+^-regulated proteins that—in mature sperm—are involved in flagellar bending.

## Author Summary

New life begins with the fusion of a sperm and an oocyte. During fertilization, sperm cope with demanding endeavors: they have to locate the egg by directed swimming and penetrate the oocyte's vestments. Cyclic nucleotides and their targets represent one of the most ancient signaling systems and are important for the control of sperm function. Here, we report the identification and characterization of an entirely novel cyclic nucleotide-binding protein—CRIS—that is crucial for male fertility. CRIS controls sperm development and, thereby, the flagellar beat of sperm. Genetic ablation of CRIS impairs sperm motility hampering steering of sperm through the female genital tract—a deficit that severely reduces male fertility. Thus, we identified an important player in the signaling events that control fertilization, which might open new avenues for the treatment of fertility defects and the design of novel contraceptives.

## Introduction

Cyclic nucleotide-binding proteins play a prominent role in cellular signaling. Their activity is regulated by binding of cAMP and/or cGMP to a highly conserved cyclic nucleotide-binding domain (CNBD), consisting of eight beta sheets that are flanked by three alpha helices [1]. Six different members of the CNBD-containing protein family have been identified: cyclic nucleotide-sensitive ion channels (CNG channels: CNGA1-4, CNGB1, 3; HCN channels: HCN1-4) [2], [3], protein kinase A and G (PKA, PKG) [4], [5], a guanine nucleotide-exchange factor (Epac1-2) [6], [7], and a bacterial transcription factor (CAP) [8]. Although the binding of ligand is conveyed to different effector domains like a catalytic kinase domain or a channel gate [9], the CNBD is highly conserved in all those proteins.

Here, we report on the identification and functional characterization of an entirely novel member of the CNBD-containing protein family, which we call CRIS (cyclic nucleotide receptor involved in sperm function). We show that CRIS controls sperm function and, thereby, male fertility.

## Results

### CRIS is a novel CNBD-containing protein

**Figure 1:**
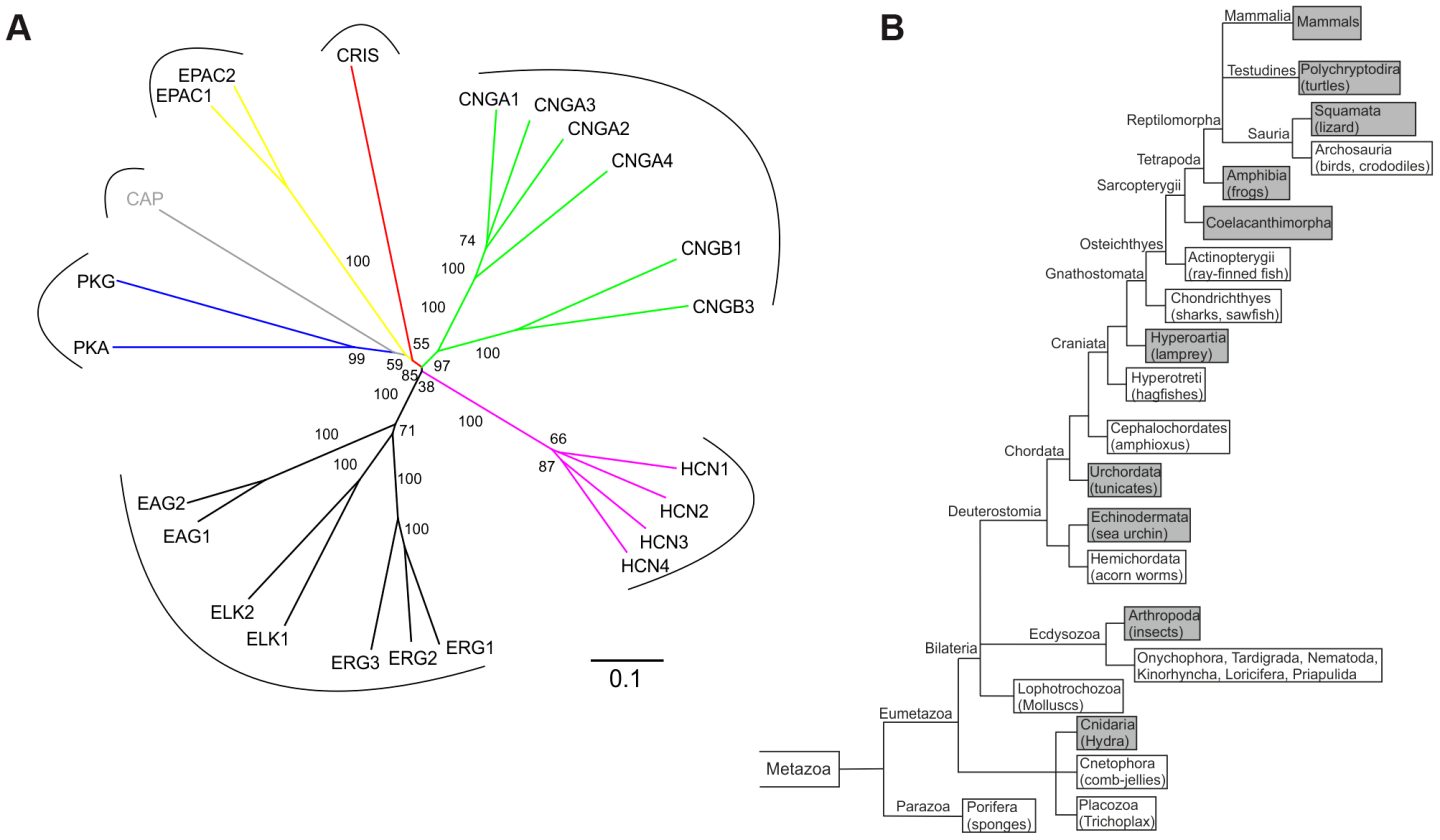
CRIS constitutes a new member of the CNBD-containing protein family. (**A**) Phylogenetic tree of CNBD-containing proteins. The different families have been labeled with different colors. Bootstrap values are shown as percentages. Scale bar shows amino acid substitution rate for the NJ (neighbor joining) tree. (**B**) Metazoan phylogeny describing the presence or absence of CRIS in metazoan genomes. The phylogenetic branching pattern was extracted from the Tree of Life project (http://www.toolweb.org/tree/) as of December 2012. The metazoan lineages known to contain CRIS are indicated by grey boxes, whereas those lineages that are believed to lack CRIS are indicated with white boxes.

A database search identified a cDNA sequence encoding an unknown protein with a CNBD motif. The CNBD is the only functional motif – otherwise, the protein shows no sequence or structural similarity to any other protein. We named the protein CRIS (cyclic nucleotide receptor involved in sperm function). CRIS constitutes a new member within the family of CNBD-containing proteins (Figure 1A). According to the NCBI and Ensembl database, orthologs have been annotated for 52 species (December 2012). We performed a phylogenetic analysis to learn about the evolutionary conservation of CRIS orthologs. CRIS has been identified in all published mammalian genomes (Fig. 1B). Moreover, CRIS exists in some amphibians and reptiles (lizards, softshell turtles, clawed frogs), jawless fish (lampreys), fish-like marine chordata (*Branchiostoma*), cnidaria (*Hydra*), tunicates (*Ciona*), echinodermata (*Strongylocentrotus*), and insects (*Drosophila*). CRIS is lacking in birds, fish (e.g. zebrafish), mollusks, nematodes (e.g. *C.elegans*), and a diverse selection of protostomes.

**Figure 2:**
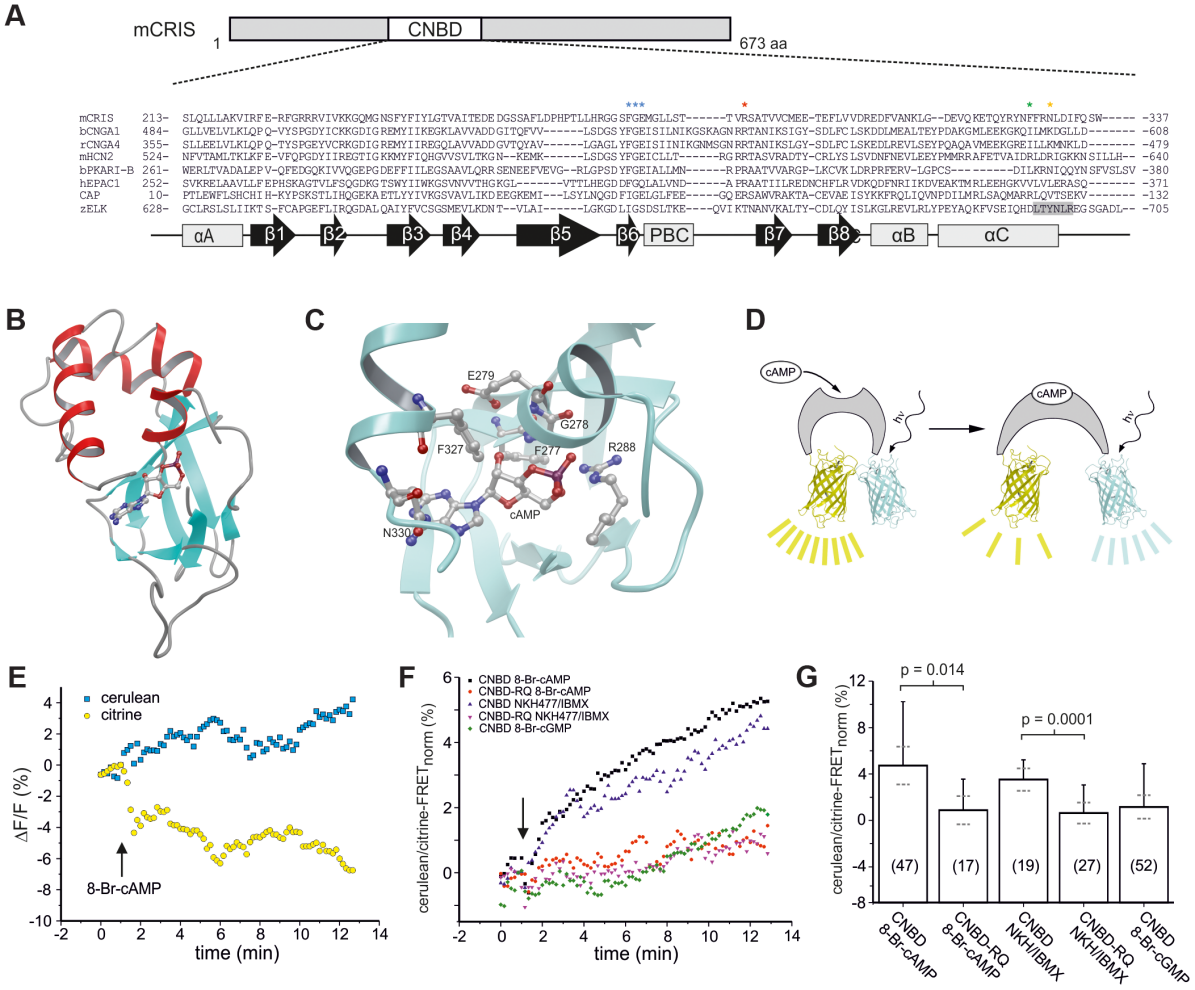
CRIS is a novel target for cAMP. (**A**) Sequence comparison of CNBDs from different proteins. Sequence alignment of CNBDs from mCRIS, cyclic nucleotide-gated channels (bCNGA1, rCNGA4), a hyperpolarization activated and cyclic nucleotide-gated channel (mHCN2), a regulatory subunit from PKA (bPKARI-B), the exchange protein directly-activated by cAMP (hEPAC1), the bacterial catabolite activator protein (CAP), and the ELK1 channel from zebrafish (zELK). Amino acids that have been shown to be essential for ligand binding [1] are highlighted with asterisks. The β strand that functions as an intrinsic ligand in the ELK channels is highlighted in grey. Secondary structure elements are indicated below (β sheets: β 1–8, black arrows; α helices: αA–C, PBC, grey boxes). (**B**) M4T model of the presumed CNBD of mCRIS in the presence of cAMP. (**C**) Close-up view of the CNBD model of mCRIS indicating important interactions of side chain and backbone atoms with cAMP. (**D–G**) Analysis of cAMP binding using FRET. (**D**) Model demonstrating that binding of cAMP changes the conformation of the CNBD resulting in a change in FRET. (**E**) Representative traces for the change in cerulean (blue) and citrine (yellow) emission during perfusion of cit-mCNBD-cer expressing CHO cells with 3 mM 8-Br-cAMP. Arrow indicates start of perfusion. (**F**) Average change in FRET (normalized emission ratio cerulean/FRET-citrine) during perfusion of cit-mCNBD-cer expressing cells with 3 mM 8-Br-cAMP (CNBD 8-Br-cAMP), 40 µM NKH477/100 µM IBMX (CNBD NKH/IBMX), 3 mM 8-Br-cGMP (CNBD 8-Br-cGMP), and cit-mCNBD-R288Q-cer expressing cells with 3 mM 8-Br-cAMP (CNBD-RQ 8-Br-cAMP), and 40 µM NKH477/100 µM IBMX (CNBD-RQ NKH/IBMX). Arrow indicates start of perfusion. (**G**) Average change in FRET after 10 min of perfusion (mean ± s.d., black; 95% confident interval, dotted, grey). N numbers and p values are indicated.

The CNBD of CRIS is characterized by the typical sequence motif of other CNBDs [1]: it is ∼120 residues long and comprises eight beta strands and three alpha helices. The beta strands form a beta barrel and harbor a phosphate binding-cassette (PBC, Figure 2A). To identify commonalities and differences between the CNBD of CRIS and other cyclic nucleotide-regulated proteins, we built a structural model based on the CNBDs of a sea urchin HCN channel (SpIH, 2ptm) [10] and Epac2 (3cf6) [11] (Figure 2B). In this model, we studied the interaction of cAMP with side chain and backbone atoms of the polypeptide. A characteristic Arg residue (R288) in the PBC is in close contact with the negatively charged exocyclic phosphate of cAMP (Figure 2A, red asterisk, Figure 2C). Furthermore, a highly conserved Phe-Gly-Glu (F277/G278/E279) motif in β6 interacts with the 2′-OH ribose moiety of cAMP (Figure 2A, blue asterisks, Figure 2C). Finally, two interactions occur between αC-helix residues and the purine ring of cAMP, one involving F327 (Figure 2C, green asterisk) and another involving N330 (Figure 2C, orange asterisk).

High-affinity CNBDs, like those of HCN channels [12] and of a bacterial CNG channel [13], bear an Arg residue at the respective position of N330 [12], [14]. Replacement of this Arg by Ala strongly lowers the ligand affinity of a CNG channel from *Mesorhizobium loti* (mlCNG) [14], [15], suggesting that this Arg residue sets apart high- from low-affinity CNBDs [1]. In this respect, the CNBD of CRIS probably represents a low-affinity binding domain, like in Epac and mammalian CNG channels, that binds cyclic nucleotides in the micromolar range of concentrations [1], [16], [17]. In summary, the protein-ligand interactions predicted by the CRIS model are similar to those of classical CNBDs, suggesting that CRIS binds cyclic nucleotides.

Of note, the KCNH channel family (EAG, ERG, ELK) carry a CNBD that, however, does not bind cyclic nucleotides [18]. The KCNH family members share a conserved sequence motif C-terminal of the αC-helix (LTYNLR in zELK, grey box, Figure 2A); the motif forms a β strand that occupies the binding pocket, suggesting that it serves as an auto-ligand for the channel [18]. However, this motif is absent in all CRIS orthologs, indicating that the CNBD represents a functional CNBD.

We experimentally studied binding of cyclic nucleotides to the CNBD using Förster resonance energy-transfer (FRET). The FRET sensor contained the CNBD from mouse CRIS (mCRIS, accession number JN629039) sandwiched between the FRET pair citrine and cerulean (cit-mCNBD-cer, Figure 2D). Similar FRET constructs using CNBDs of other proteins, e.g. Epac, have been successfully employed to detect binding of cyclic nucleotides [19]–[24]. When expressed in HEK293 cells, cit-mCNBD-cer displayed a FRET signal. However, the intracellular distribution was not uniform among cells. In some cells, the FRET sensor was clustered, whereas in other cells, it showed a rather homogenous distribution. In the latter, the FRET signal depended on the intracellular concentration of cyclic nucleotides (Figure 2E). Addition of 8-Br-cAMP, a membrane-permeable cAMP analogue, or NKH477, an activator of adenylyl cyclases, changed the ratio of the cerulean/citrine-FRET: the fluorescence of the acceptor (citrine) was diminished, whereas the fluorescence of the donor (cerulean) was increased (Figure 2E–G). In contrast, 8-Br-cGMP did not change FRET (Figure 2F, G). A mutant construct (cit-mCNBD-R288Q-cer FRET), in which ligand binding was impaired by mutating the conserved arginine in the PBC (R288Q) [25], [26], was rather uniformly distribute throughout the cell, but did not respond to changes in cAMP (Figure 2F, G). These results indicate that CRIS, in fact, is a cyclic nucleotide-binding protein with a preference for cAMP.

### CRIS is exclusively expressed in spermatocytes and round spermatids

**Figure 3:**
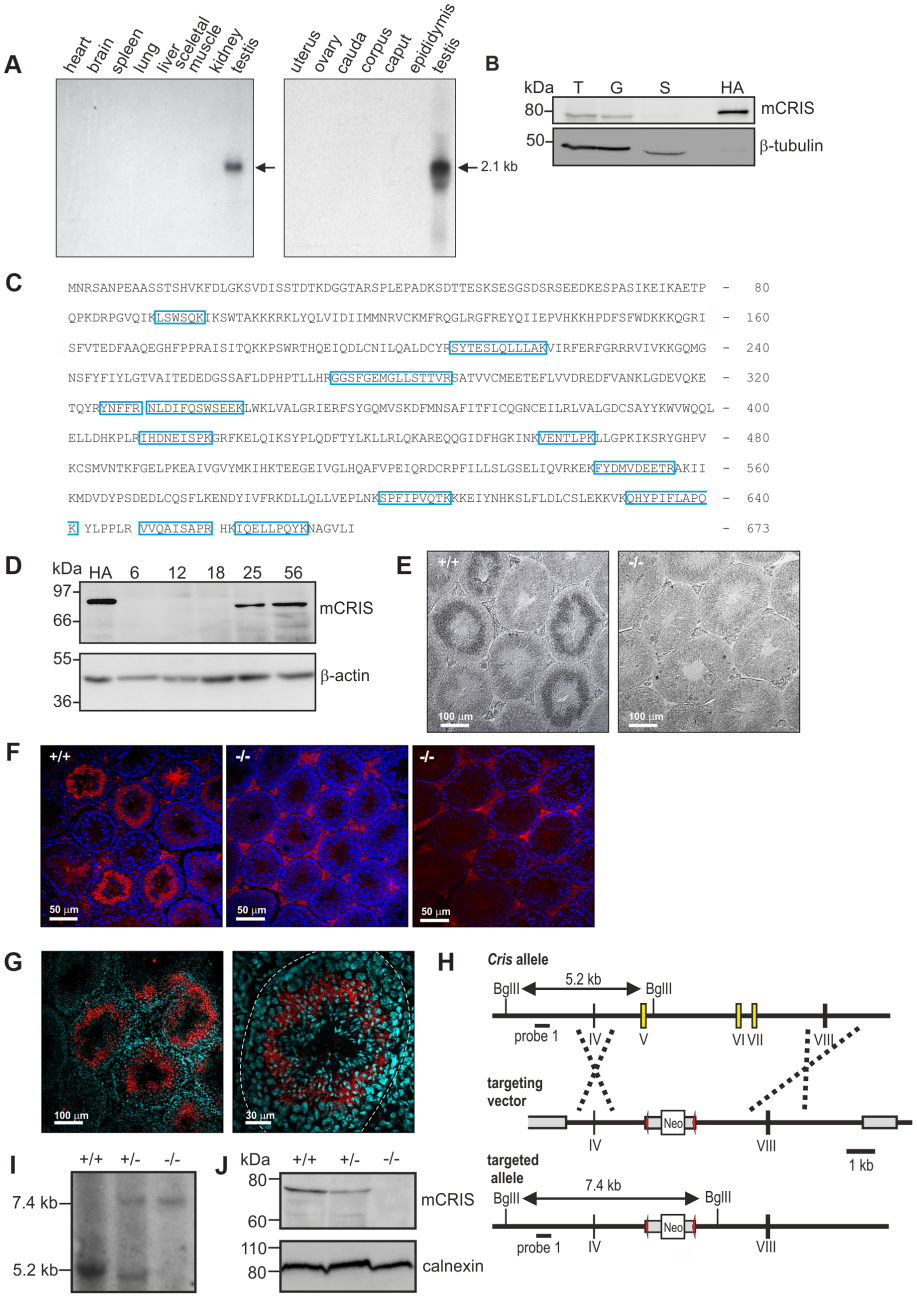
CRIS is exclusively expressed in sperm precursor-cells. (**A**) Analysis of *Cris* mRNA expression by Northern blot. Left, mouse multi-tissue; right, mouse reproductive tissue. (**B**) Analysis of CRIS protein expression by immunoblotting using a CRIS-specific polyclonal antibody. Protein lysates: T, testis (50 µg); G, germ cells (50 µg); S, cauda sperm (100 µg); HA, mCRIS-HA expressing HEK293 cells (15 µg). Loading control: β-tubulin. (**C**) Identification of mCRIS in testis using mass spectrometry. Testis lysates were separated on a 1D SDS-PAGE, lanes were sliced, and analyzed by mass spectrometry. Unique peptides for mCRIS are indicated in blue. (**D**) Developmental expression pattern of mCRIS in testis. Proteins from mouse testis (30 µg/lane) have been probed with a CRIS-specific monoclonal antibody. The age of the mice (days after birth) is indicated. Control: mCRIS-HA expressing HEK cells (10 µg/lane); loading control: β-actin. (**E**) *In situ* hybridization. Testis sections (+/+: wild-type, −/−: CRIS knockout) have been labeled with a *Cris*-specific anti-sense probe. Dark staining indicates a positive signal. The corresponding sense probe showed no staining. Scale bars are indicated. (**F**) Immunohistochemical analysis of CRIS in mouse testis. Testis sections (+/+: wild-type, −/−: CRIS knockout) have been probed with a polyclonal CRIS-specific antibody and a fluorescent secondary antibody (red). DNA was stained with DAPI (blue). The secondary antibody unspecifically labels the interstitial cells in between the tubules (see −/−, right, secondary antibody only). Scale bars are indicated. (**G**) See (F) Higher magnification; dotted line: *lamina propria*. (**H**) Targeting strategy for the generation of *Cris*-deficient mice. Exons 5–7 (yellow boxes) have been replaced with a neomycin cassette (Neo) flanked by two lox-P elements (red arrow heads). Restriction sites, the corresponding fragment sizes, and the localization of probe 1 are indicated. (**I–J**) Verification of gene targeting. (**I**) Southern blot analysis using probe 1. The sizes of the fragments are indicated. (**J**) Analysis of CRIS expression in germ cells using a polyclonal CRIS-specific antibody (50 µg/lane). Loading control: calnexin.

To unravel the physiological function of CRIS *in vivo*, we determined the expression pattern of mouse CRIS (mCRIS) by Northern blot, *in situ* hybridization, Western blot, immunohistochemistry, and mass spectrometry. Northern blot analysis using mRNAs from different tissues revealed that *Cris* mRNA is only transcribed in testis (Figure 3A). In a similar vein, CRIS protein was detected by different polyclonal and monoclonal antibodies only in immunoblots from lysates of testis. In particular, CRIS was present in precursor cells, but not in cauda sperm from the epididymis (Figure 3B). To verify these results, we performed mass spectrometry. Protein lysates were separated on a 1D gel (SDS-PAGE), lanes were sliced, and analyzed by mass spectrometry. We identified 12 peptides distributed over the entire sequence of CRIS in protein lysates from testis, but not from cauda sperm (Figure 3C).

During development, CRIS was detected after day P18 (Figure 3D), i.e. when the first haploid cells – the secondary spermatocytes - emerge. To analyze when CRIS expression starts and ends, we performed *in situ* hybridization and immunohistochemistry on testis sections: *mCris* mRNA was expressed in spermatocytes (Figure 3E) and mCRIS protein in late spermatocytes and round spermatids (Figure 3F, G). The distribution of the mCRIS protein within cells is largely uniform, suggesting that CRIS is a cytosolic protein (Figure 3G). The expression of CRIS in certain stages during sperm development and not in mature sperm suggests that CRIS is involved in spermiogenesis, the process that involves the major morphological and function changes during spermatogenesis.

### CRIS^−/−^ males are subfertile

To study the function of CRIS *in vivo*, we generated *Cris*-deficient mice (CRIS^−/−^) by homologous recombination in embryonic stem (ES) cells. Exons 5–7, encoding the CNBD, were replaced with a neomycin resistance-cassette (Figure 3H). Disruption of the gene was confirmed by *in situ* hybridization (Figure 3E), immunohistochemistry (Figure 3F), Southern blotting (Figure 3I), and immunoblotting (Figure 3J).

The offspring of heterozygous matings exhibited roughly Mendelian proportions (wild-type (+/+): 33%, heterozygous (+/−): 40%, mutant (−/−): 27%; n = 233), demonstrating that loss of CRIS does not affect embryonic development. CRIS^−/−^ mice are indistinguishable from wild-type and heterozygous littermates regarding appearance, general behavior, and survival rate.

**Figure 4:**
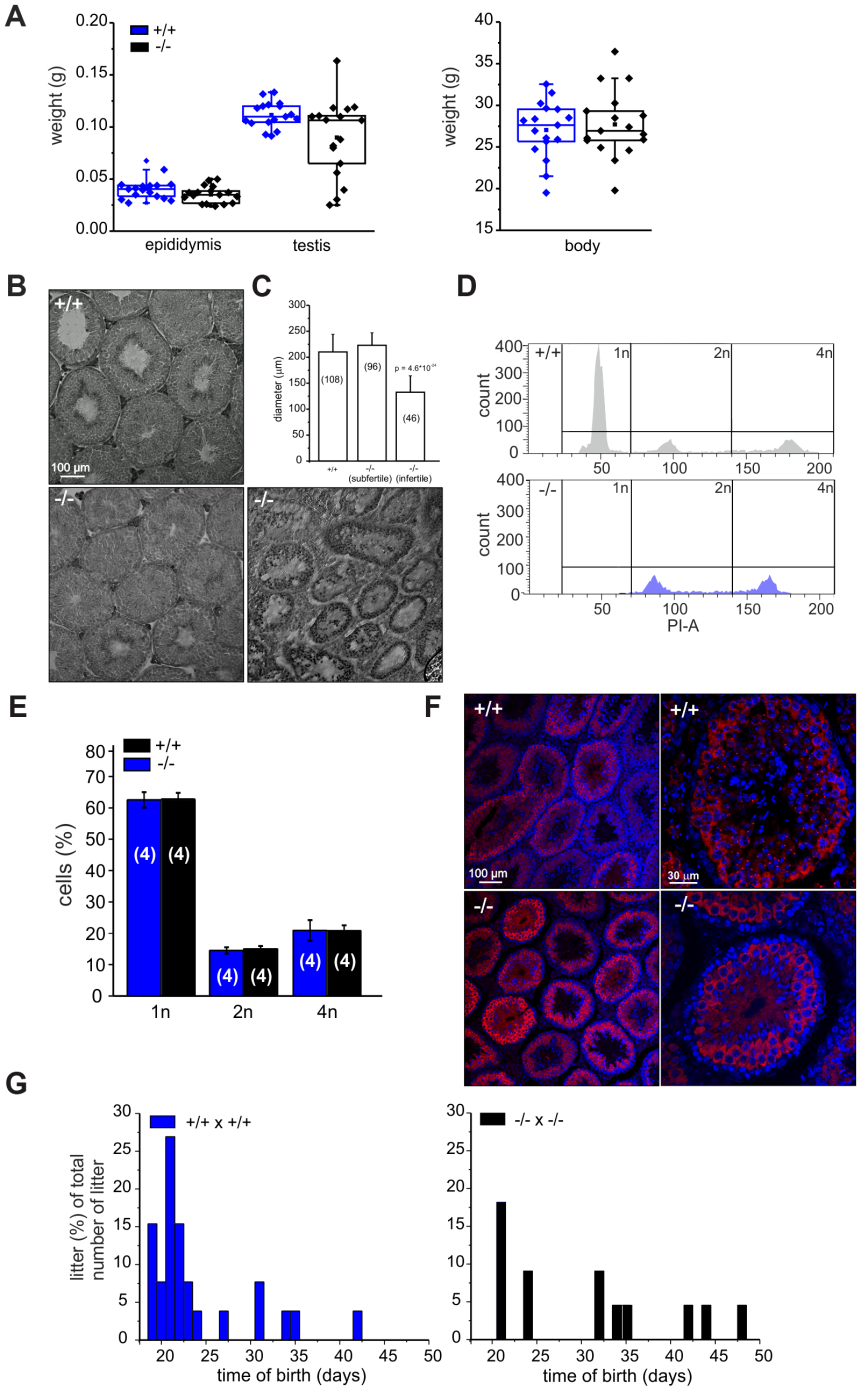
*Cris*-deficient mice are prone to spermatogenic arrest. (**A**) Testis, epididymis, and body weight of wild-type (+/+) and knockout mice (−/−). (**B**) Analysis of testis morphology using Periodic acid-Schiff staining (PAS) of frozen testis sections. Left: wild-type testis, middle: knockout (−/−) testis with normal testis weight, right: knockout (−/−) testis with lower testis weight. Scale bars are indicated. (**C**) Analysis of tubule diameter wild-type (+/+) and knockout mice (−/−) with either normal testis weight (subfertile) or lower testis weight (infertile). Number of tubules are indicated. (**D–E**) DNA-content analysis of male germ cells. (**D**) Representative histogram of propidium iodide-stained wild-type (+/+, top) and knockout (−/−, bottom, with lower testis weight) male germ cells analyzed by FACS. (**E**) Average data (mean ± s.d) of DNA content analysis from wild-type (+/+) and knockout (−/−) male germ cells with normal testis weight. Number of animals are indicated. (**F**) Immunohistochemical analysis of MIWI in mouse testis. Testis sections (+/+: wild-type, −/−: CRIS knockout with normal testis weight) have been probed with a polyclonal MIWI-specific antibody and a fluorescent secondary antibody (red). DNA was stained with DAPI (blue). Pictures on the right show a higher magnification of a single tubule. Scale bars are indicated. (**G**) Breeding performance of wild-type (left, blue) and knockout males (right, black) mated with wild-type females. Days between first mating and birth of litter are plotted. The number of litters per day is expressed as a percentage of the total number of litters.

Because CRIS is exclusively expressed in testis, we determined testis and epididymis weight of wild-type and mutant males. Whereas epididymis weight was similar, testis weight in mutant males was highly variable compared to wild-type males (Figure 4A): 19% of mutant testes were significantly smaller. Males with reduced testis weight had no cauda sperm and testis morphology was different: the organization of the tubules was altered and no sperm were present in the lumen (Figure 4B). The testis morphology of knockout males with normal testis weight was similar to wild-type testis (Figure 4B). Next, we determined the diameter of the seminiferous tubules from wild-type and knockout testis. Whereas wild-type testis and testis from knockout males with normal testis weight were comparable, the diameter of tubules from knockout males with lower testis weight was reduced by ∼50% (Figure 4C). This correlates well with the lower testis weight observed for those animals.

We hypothesized that in knockout males with lower testis weight, spermatogenesis was arrested. Therefore, we analyzed the DNA content of male germ cells using flow cytometry to determine the relative distribution of haploid (1n), diploid (2n), and tetraploid (4n) cells (Figure 4D, E). Mutant testis with lower weight contained only tetraploid and diploid, but no haploid cells (Figure 4D, bottom). The first haploid cells are the secondary spermatocytes. Thus, spermatogenesis in a group of *Cris*-deficient males stops before the meiotic division and, thereby, avoids the generation of haploid cells, rendering those males infertile.

In contrast, mutant males with normal testis weight displayed a distribution of haploid (1n), diploid (2n), and tetraploid (4n) cells similar to wild-type testis (Figure 4E). We analyzed spermatogenesis in more detail using immunohistochemistry. Since spermatogenesis in infertile knockout males stops before the generation of secondary spermatocytes, we investigated whether the transition from primary to secondary spermatocytes proceeds normally in knockout males with normal testis weight. We labeled testis sections with an antibody against MIWI, a piRNA-interacting protein that controls spermatogenesis and is expressed in the later stages of primary spermatocytes, in secondary spermatocytes, and in round spermatids [27]. The staining pattern in wild-type and knockout testis with normal testis weight was similar (Figure 4F), underlining our FACS result that both contained the full set of sperm precursor cells namely spermatogonia, spermatocytes, and spermatids.

However, also knockout males with normal testis weight showed a fertility defect: only 60% (13/22, Table 1) of all wild-type females that were mated with mutant males (with normal testis weight) for four weeks became pregnant (compared to 100%, 26/26 in wild-type matings, Table 1). Females that had offspring needed a longer time to become pregnant and their litter size was smaller (Figure 4G, Table 1). The frequency of vaginal plugs was similar between matings with wild-type and mutant males, indicating that mating behavior was normal (Table 1). *In vitro* fertilization assays were performed to test the ability of mutant sperm to fertilize eggs. Wild-type or mutant sperm were incubated with wild-type oocytes and the number of two-cell stage embryos was counted after 18 h. There was no significant difference between the two genotypes regarding the percentage of two-cell stage embryos (+/+: 48±15%, 312 2-cell stages/655 oocytes; −/−: 61±25%, 481 2-cell stages/793 oocytes, data are presented as mean ± s.d.), indicating that sperm from mutant males are able to penetrate the zona pellucida, the outer layer of the egg.

**Table 1:** Phenotypic characterization of *Cris*-deficient mice.

	+/+	+/−	−/−
sperm count (million cells/ml)	12±5 (21)	11±4 (18)	9±6 (17)
plug positive after mating with +/+ females (days)	1.17±0.5 (6)	0.83±0.3 (6)	1.16±0.2 (6)
pregnant +/+ females	100% (26/26)	93.3% (14/15)	59.1% (13/22)
days between mating and birth of litter	22.4±5.3 (26)	24.1±5.8 (14)	30.5±9.5 (13) (p = 0.004 to +/+)
litter size at birth	7.9±0.4 (26)	6.9±0.5 (14) (p = 0.04 to −/−)	5.1±0.7 (13) (p = 0.0006 to +/+)

Our results show that CRIS plays an important role during sperm development. However, infertility in *Cris*-deficient males displays partial penetrance, thereby creating two groups: one with normal testis weight and morphology, which are subfertile, and one with reduced testis weight and aberrant testis morphology, which are infertile.

### CRIS determines the development of flagellar bending

**Figure 5:**
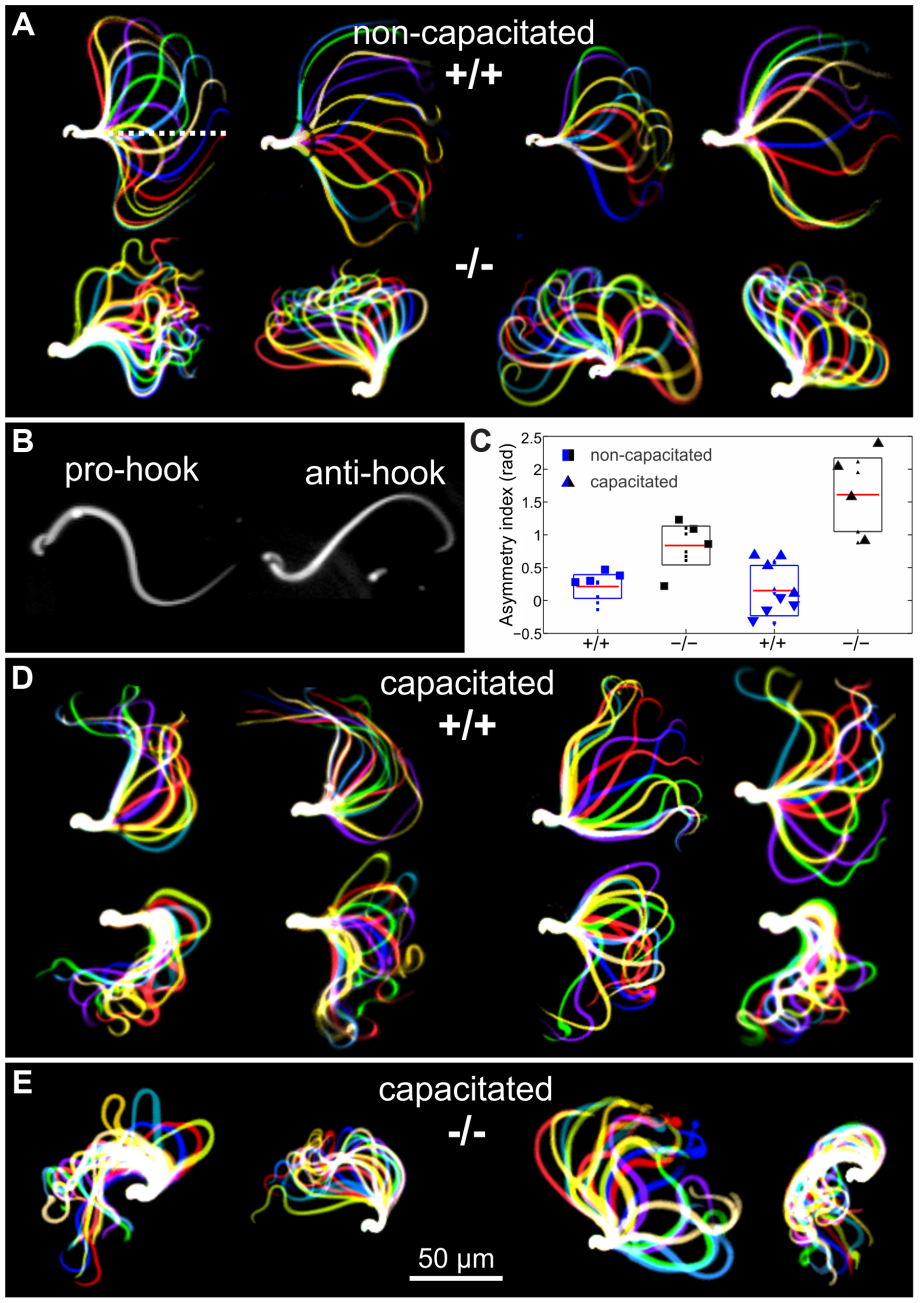
Flagellar waveform. (**A**) Representative images showing the flagellar waveform of tethered non-capacitated wild-type (+/+) and mutant (−/−) sperm. Images were acquired at 200 frames per second. (**B**) Flagellar bending modes of mouse sperm. Left: pro-hook conformation, right: anti-hook conformation. (**C**) Quantification of the flagellar-waveform asymmetry for cells shown in panels A, D, and E. The height of the boxes indicates the s.d. around the mean (red). Big symbols on each boxplot are displayed in the same order (from left to right) as the cells shown on the corresponding panel. Small symbols represent cells that are not shown in the figure. Blue triangles pointing up (down) correspond to those cells on the top (bottom) of panel D. (**D**) Capacitated wild-type sperm alternate between bending in the pro- (bottom row) and anti-hook conformation (top row). (**E**) Capacitated mutant sperm remain in the anti-hook conformation.

Sperm are propelled by bending of the flagellum [28] and fertility phenotypes often result from defects in sperm motility [29]. Although there was no evidence that CRIS is expressed in mature sperm, we considered the possibility that CRIS controls processes during sperm development that are later required for sperm motility. Therefore, we compared the flagellar beat and motility of wild-type and mutant sperm. In non-capacitated wild-type sperm, the midpiece was straight and the flagellar beat was symmetrical with respect to a line through the midpiece (Figure 5A, dotted line). In contrast, the midpiece of mutant sperm was bent, resulting in a highly asymmetrical flagellar waveform (Figure 5A). Asymmetrical beating is a hallmark of hyperactivation – a swimming behavior that assists sperm to swim through the oviductal mucus, to leave the sperm reservoir at the oviductal isthmus, and to penetrate the egg vestments [30]–[32]. Hyperactivated motility is initiated by a maturation process called capacitation [33], [34]. Mouse sperm are an excellent animal model to study the symmetry of the flagellar beat. Their head is hook-shaped, which allows determining the direction of flagellar bending. In the pro-hook bending state, the flagellum and the hook were pointing towards the same side, whereas in the anti-hook conformation, the flagellum and the hook were facing opposite sides (Figure 5B) [32], [35]. Under capacitating conditions, wild-type mouse sperm displayed a highly asymmetrical flagellar waveform (Figure 5D); flagellar bending in one and the same cell switched between the pro- (bottom row, Figure 5D) and anti-hook conformation (top row, Figure 5D). In contrast, the midpiece of mutant sperm, no matter whether non-capacitated or capacitated, always remained in the anti-hook conformation (Figure 5E). Furthermore, in capacitated mutant sperm the asymmetry of the flagellar waveform was even further enhanced (Figure 5E). We quantified the difference in flagellar bending between wild-type and knockout sperm by calculating the asymmetry index (Figure 5C). An asymmetry index of 0 indicates a symmetric flagellar beat. Bending in the anti-hook conformation is reflected by an increase, bending in the pro-hook conformation by a decrease in the asymmetry index. Under non-capacitating conditions, the asymmetry index of knockout sperm is more positive compared to wild-type sperm. Under capacitating conditions, the asymmetry index of knockout sperm is even further increased, whereas wild-type sperm display both, a positive and a negative asymmetry index, reflecting the switch between the pro- and anti-hook conformation (Figure 5C).

**Figure 6:**
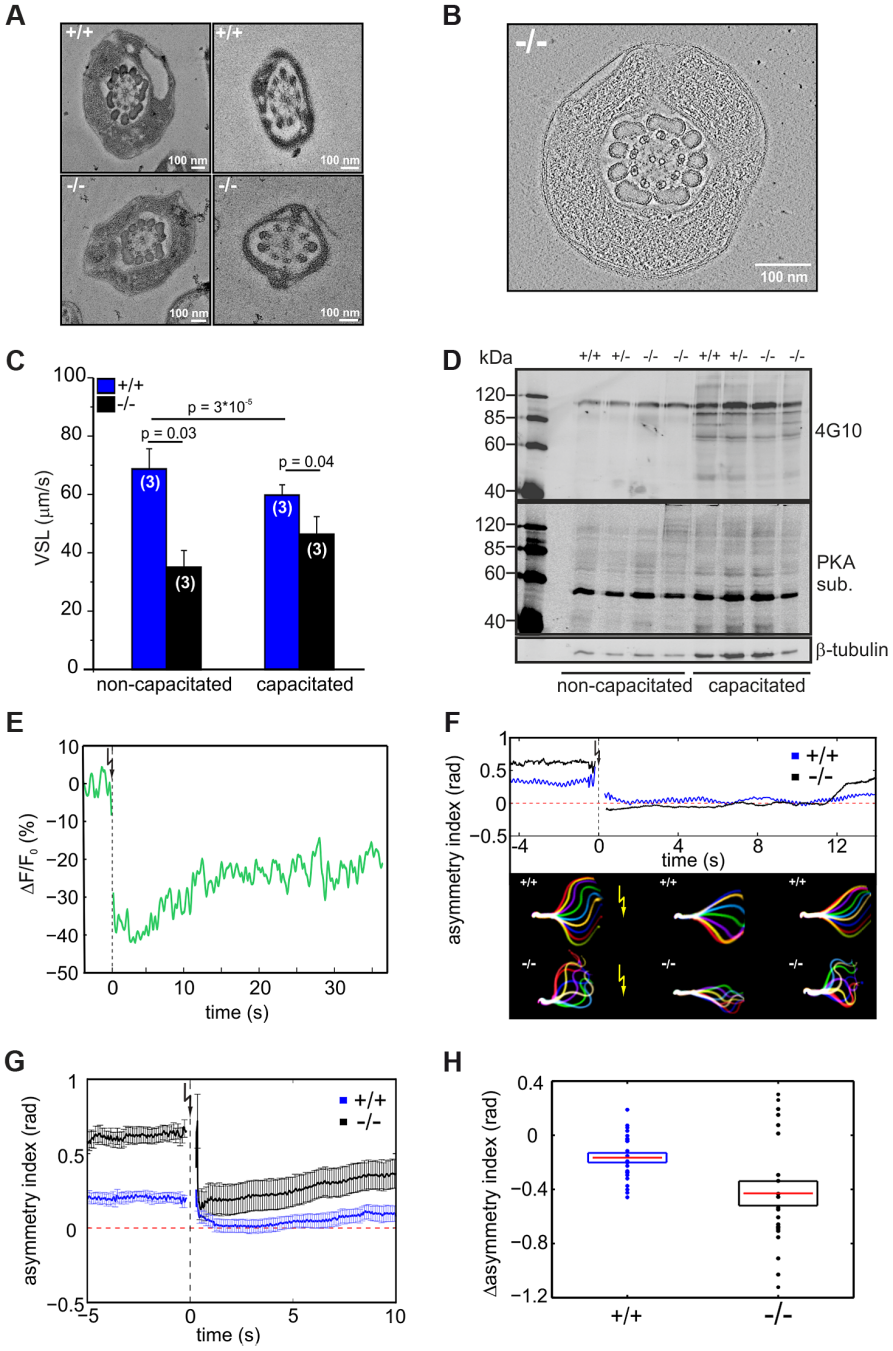
CRIS controls the development of flagellar bending. (**A**) Electron micrograph (cross section) of the midpiece (left) and principal piece (right) of the sperm flagellum from wild-type (+/+, top) and *Cris*-deficient (−/−, bottom) mice. (**B**) Minimal projection of a cross-section through the midpiece of the sperm flagellum from *Cris*-deficient (−/−) mice. The corresponding movie (Movie S1) can be found in the supplementary information. (**C**) Analysis of sperm motility. Velocity of straight line path (VSL) has been determined from the corresponding movies (Movie S2, S3). Data are presented as mean ± s.d. (**D**) Analysis of PKA- and tyrosine kinase-dependent protein phosphorylation under capacitating conditions and non-capacitating conditions. Per conditions, 1×10^6^ cells were used. Immunoblots have been probed with PKA and tyrosine kinase substrate-specific (4G10) antibodies. Loading control: β-tubulin. (**E**) Ca^2+^ imaging of mouse sperm loaded with caged 2-Diazo and Fluo-8. 2-Diazo was released using a UV flash. Shown is a representative trace of a wild-type sperm. Fluorescence has been background-subtracted and normalized to the value before the flash. (**F**) Flagellar waveform analysis of sperm from *Cris*-deficient mice loaded with caged 2-Diazo. Representative analysis showing the asymmetry index before and after the UV flash. Corresponding images of the flagellar waveform before and after the UV flash (yellow arrow) are included. (**G**) Mean values of the asymmetry index for wild-type (blue) and mutant sperm (black). Data are plotted as mean ± s.e.m. Corresponding movies (Movie S4, S5) can be found in the supplementary information (+/+: n = 24, −/−: n = 22). (**H**) Relative change of asymmetry index for wild-type (blue) and mutant sperm (black). Values after the UV flash were normalized to the values before the flash. Individual data are plotted as circles, the corresponding mean is represented by a red line, and the s.e.m. is indicated with a box, p = 0.0075.

The difference in flagellar bending between wild-type and mutant sperm could either result from a defect in the flagellar ultrastructure or in the molecular mechanisms controlling the beat. We compared the ultrastructure of wild-type and mutant sperm using electron microscopy and did not detect any major difference in the 9+2 microtubule structure of the axoneme or any other morphological anomalies (Figure 6A, B, Movie S1).

To investigate how the defect in flagellar bending affects sperm motility, we studied the swimming behavior of wild-type and mutant sperm under non-capacitating and capacitating conditions. The swimming behavior can be characterized by the velocity of straight-line path (VSL), which is a measure for progressive swimming: an increase of VSL reflects an increase in progressive motility [36]. The VSL of mutant sperm was lower than that of wild-type sperm under both non-capacitating (Movie S2; Figure 6C, +/+: 67±7 µm/s; −/−: 35±6 µm/s; number of animals: n = 3) and capacitating conditions (Movie S3; Figure 6C, +/+: 56±4 µm/s; −/−: 46±6 µm/s, n = 3). Thus, *Cris*-deficient sperm swim less progressively than wild-type sperm.

We tested whether the asymmetric beat of mutant sperm results from premature capacitation. A hallmark of sperm undergoing capacitation is a characteristic phosphorylation pattern evoked by PKA and tyrosine kinases [37]–[39]. However, there was no difference between the phosphorylation pattern of wild-type and knockout sperm under both non-capacitating and capacitating condition, indicating that the asymmetric beating of knockout sperm does not result from premature capacitation (Figure 6D).

Ca^2+^ plays an important role in sperm motility. It controls chemotactic steering of invertebrate sperm [40]–[42] and Ca^2+^ entry through CatSper channels promotes the transition to hyperactivated motility [43], [44]. In this respect, the asymmetric flagellar beat of mutant sperm recapitulates the action of an elevated intracellular Ca^2+^ concentration [Ca^2+^]_i_. However, [Ca^2+^]_i_ between wild-type sperm and sperm from *Cris*-deficient mice under non-capacitating conditions was not different (+/+: 483±34 nM, −/−: 478±24 nM; number of animals: n = 3). Thus, we hypothesized that the Ca^2+^ regulation of the flagellar beat rather than [Ca^2+^]_i_ is altered in CRIS knockout sperm. Therefore, we studied the flagellar beat of wild-type and mutant sperm exposed to a controlled change in [Ca^2+^]_i_. Sperm were incubated with a cell-permeant, photoactivatable Ca^2+^ scavenger (2-Diazo) that rapidly binds free Ca^2+^ upon flash photolysis [45]. After the UV flash, [Ca^2+^]_i_ was lower (Figure 6E) and sperm responded with a change in flagellar waveform (Movie S4, S5). We quantified the change in flagellar bending using the asymmetry index (Figure 6F, G). One second before the UV flash, mutant sperm displayed a higher asymmetry index than wild-type sperm (Figure 6G). One second after the flash, the beat of both wild-type and mutant sperm became more symmetrical (Figure 6G). However, the action of lower [Ca^2+^]_i_ was more dramatic in mutant sperm, which displayed a significantly larger change in asymmetry than wild-type sperm (Figure 6G, H). Thus, the flagellar beat of mutant sperm is not irreversibly locked in the asymmetric state (i.e. anti-hook mode) and is also dependent on [Ca^2+^]_i_. In fact, the asymmetry index of mutant sperm at low [Ca^2+^]_i_ was similar to that of wild-type sperm at resting [Ca^2+^]_i_ (Figure 6G). This result argues for an altered Ca^2+^ regulation of the flagellar beat in sperm from *Cris*-deficient mice.

A target for Ca^2+^ in the sperm flagellum that has been suggested to control sperm motility is the Ca^2+^/calmodulin-dependent kinase IV (CaMKIV) [46]. To investigate the effect of CaMKIV activity on flagellar bending, we incubated wild-type sperm with 10 µM KN93, an inhibitor of CaMKIV/CaMKII. KN93 did not change the amplitude or frequency of the flagellar beat (amplitude: 31±5 µm (control) vs. 24±10 µm (KN93), frequency: 6±2 Hz (control) vs. 7±3 Hz (KN93), n = 9). However, the asymmetry index of the flagellar beat changed significantly from 0.26±0.17 (control) to 0.03±0.2 (KN93, p = 0.02, n = 9), demonstrating that indeed, CaMKIV is involved in the tuning of the flagellar beat asymmetry. However, the drug decreased the asymmetry in wild-type sperm whereas in *Cris*-deficient mice, the asymmetry is increased. Thus, phosphorylation of proteins by CaMKIV rather augments the asymmetry of flagellar bending, whereas the action of CRIS seems to diminish the asymmetry of flagellar bending.

### Putative interaction partners of CRIS

Because we have no evidence that CRIS is expressed in mature sperm, CRIS cannot be directly involved in regulating the Ca^2+^ sensitivity of flagellar bending. Instead, CRIS expression during spermatogenesis suggests that during sperm development, CRIS might interact with proteins that control flagellar bending in mature sperm. We performed co-immunoprecipitation studies with a CRIS-specific antibody and subsequent mass spectrometry to identify putative binding partners of CRIS in sperm precursor cells. Similar experiments with proteins from sperm precursor-cells of *Cris*-deficient mice served as a negative control. We identified 27 candidate proteins that fall into three major groups: proteins involved in gene regulation, in flagellar transport, and in cellular signaling (Table 2). During spermatogenesis, gene transcription ceases before mature sperm are formed. A tight control of the temporal and stage-specific gene expression is a prerequisite for the correct differentiation of spermatids into mature sperm [47]. CRIS is expressed in round spermatids. Thus, CRIS might interact with proteins that are involved in regulating gene expression just before transcription stops.

**Table 2:** Proteins identified by mass spectrometry.

	Protein symbol	Accession number	Protein name	Number of peptides in +/+	Number of peptides in −/−
**Intracellular transport**	KIF2A	NP_032468.2	Kinesin-like protein KIF2A	9	0
	IFT172	NP_080574.5	Intraflagellar transport protein 172 homolog	7	0
	ABCF2	NP_038881.1	ATP-binding cassette sub-family F member 2	6	0
	SNX4	NP_542124.1	Sorting nexin-4	5	0
	DCTN4	NP_080578.1	Dynactin subunit 4	4	0
**Cellular signaling**	IQCB1	NP_796102.2	IQ calmodulin-binding motif-containing protein 1	5	0
	PKN1	NP_796236.2	Serine/threonine-protein kinase N1	5	0
	APLL1	NP_660256.1	DCC-interacting protein 13-alpha	5	0
	ASPSCR1	NP_081153.1	Tether containing UBX domain for GLUT4	5	0
	PP2BA	NP_032939.1	Serine/threonine-protein phosphatase 2B catalytic subunit alpha isoform	5	0
	KCC4	NP_035520.1	Calcium/calmodulin-dependent protein kinase type IV	4	0
	RAB5C	NP_077776.2	Ras-related protein Rab-5C	3	0
	RNF123	NP_115932.1	E3 ubiquitin-protein ligase RNF123	3	0
	OSBP1	NP_002547.1	Oxysterol-binding protein 1	3	0
	ALDH1A2	NP_033048.2	Retinal dehydrogenase 2	3	0
	OSB11	NP_789810.2	Oxysterol-binding protein-related protein 11	3	0
	JNK1	NP_057909.1	Mitogen-activated protein kinase 8	3	0
**Regulation of gene expression**	RFC4	NP_663455.1	Ribosome-binding protein 1	7	0
	RRBP1	NP_077243.2	Replication factor C subunit 4	6	0
	SELW	NP_033182.1	Selenoprotein W	4	0
	CYFIP1	NP_035500.2	Cytoplasmic FMR1-interacting protein 1	4	0
	CNOT10	NP_705813.2	CCR4-NOT transcription complex subunit 10	4	0
	PDS5B	NP_780519.3	Sister chromatid cohesion protein PDS5 homolog B	4	0
	MED17	NP_659182.1	Mediator of RNA polymerase II transcription subunit 17	4	0
	U520	NP_067359.2	U5 small nuclear ribonucleoprotein 200 kDa helicase	3	0
	PIH1D1	NP_083682.1	PIH1 domain-containing protein 1	3	0
	KTI12	NP_083847.1	Protein KTI12 homolog	3	0

Another group comprises proteins involved in flagellar transport; the top candidates being a kinesin-like protein KIF2A and the intraflagellar transport protein IFT172 (Table 2). In *Chlamydomonas*, proteins involved in intraflagellar transport (IFT) carry cargo between the cytoplasmic basal body and the assembly site at the distal tip of the cilium [48], [49]. In sperm, these mechanisms are poorly understood. ABCF2 is a cytosolic member of the ABC transporter family that has been shown to control cell volume [50]. The putative interaction with IFT172, KIF2A, and ABCF2 in sperm precursor cells suggests that CRIS regulates protein transport into the flagellum during spermiogenesis.

**Figure 7:**
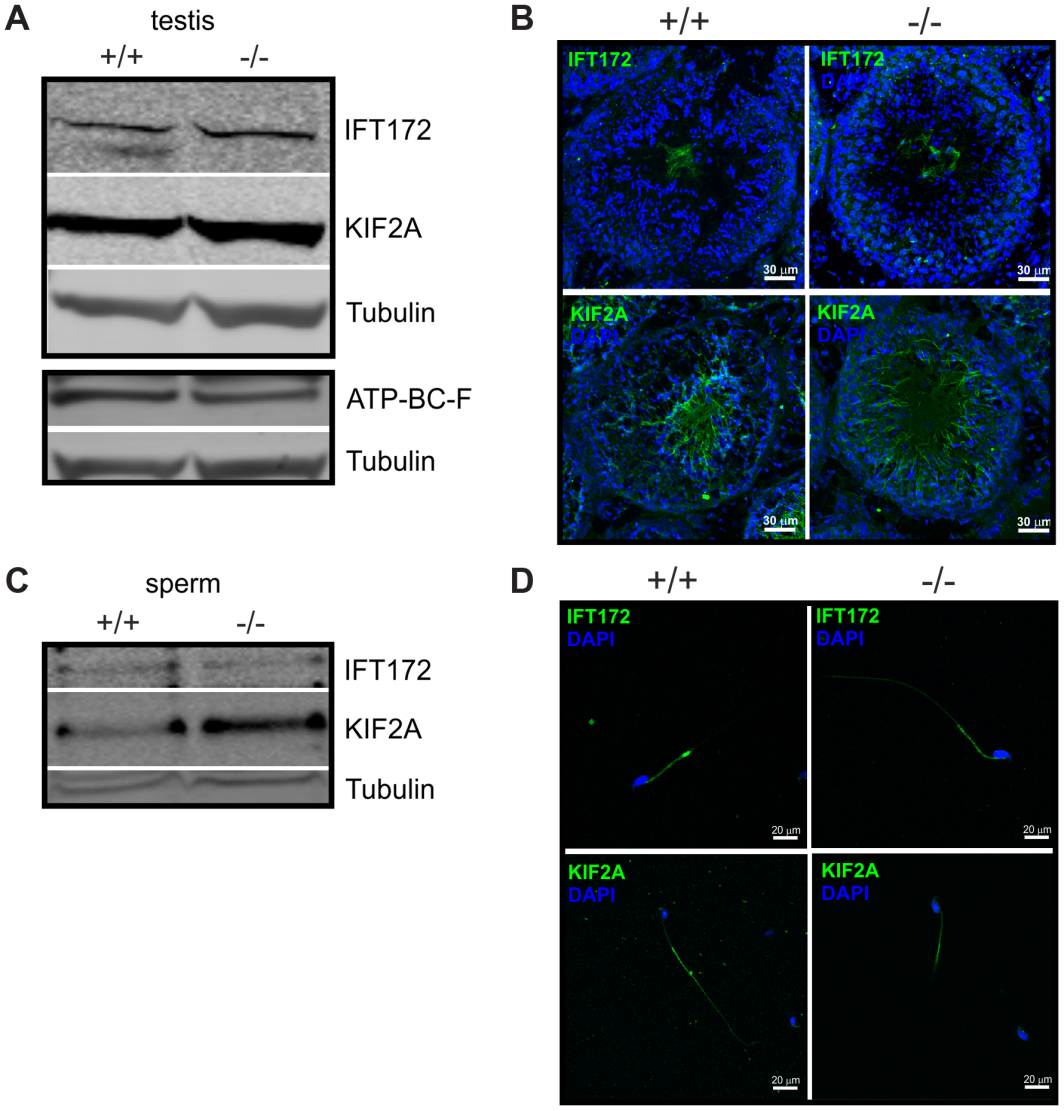
Expression of putative CRIS interaction partners. (**A**) Analysis of protein expression in testis from wild-type (+/+) and *Cris*-deficient mice (−/−) by immunoblotting. Per lane, 50 µg total testis lysates have been loaded. Antibodies against KIF2A, IFT172, and ABCF2 are described in Table 4. Loading control: β-tubulin. (**B**) Immunohistochemical analysis of IFT172 and KIF2A in mouse testis. Testis sections (+/+: wild-type, −/−: CRIS knockout) have been probed with IFT2- and KIF2A-specific antibodies and a fluorescent secondary antibody (green). DNA was stained with DAPI (blue). Scale bars are indicated. (**C**) Analysis of protein expression in sperm from wild-type (+/+) and *Cris*-deficient mice (−/−) by immunoblotting. Per lane, total protein from 5×10^6^ cells has been loaded. Loading control: β-tubulin. (**D**) Immunocytochemical analysis of IFT172 and KIF2A in mouse sperm. Sperm isolated from the cauda (+/+: wild-type, −/−: CRIS knockout) have been probed with IFT172- and KIF2A-specific antibodies and a fluorescent secondary antibody (green). DNA was stained with DAPI (blue). Scale bars are indicated.

We analyzed the expression pattern of IFT172, KIF2A, and ABCF2 in testis and sperm from wild-type and *Cris*-deficient mice (Figure 7). In testis lysates, we could detect all three proteins. However, there was no obvious difference in expression between wild-type and knockout mice (Figure 7A). Immunohistochemical analysis of testis sections revealed that IFT172 and KIF2A show a punctuated staining in round spermatids, but are predominantly expressed in the flagellum of elongated spermatids and sperm in the testis lumen. However, there was no difference in the staining pattern between wild-type and knockout mice (Figure 7B). The antibody against ABCF2 did not show any specific labeling on testis sections (data not shown). IFT172 and KIF2A were both detected on a Western blot using total sperm lysates, but again, there was no obvious difference between genotypes (Figure 7C). Immunocytochemical analysis of isolated cauda sperm revealed that IFT172 is localized in the midpiece and cytoplasmic droplet, whereas KIF2A is predominantly localized in the principal piece (Figure 7D). However, the staining pattern was similar for wild-type and *Cris*-deficient mice. Again, the ABCF2 antibody did not show any specific labeling (data not shown). Thus, the overall expression of these putative interaction partners seems to be similar in wild-type and *Cris*-deficient mice. Also, their localization pattern, analyzed by confocal microscopy, showed no major difference between genotypes. However, it cannot be excluded that these proteins are only slightly mislocalized in the flagellum or a specific interaction with other proteins is missing.

The third group encompasses proteins that are involved in cellular signaling. At least two of these proteins - IQCB1 (IQ motif containing B1) and CaMKIV (Ca^2+^/calmodulin-dependent protein kinase IV) - have been associated with a flagellar function (Table 2). IQCB1 harbors an IQ calmodulin-binding motif; the protein has been linked to retinal and renal ciliopathy [51], [52]. CaMKIV has been proposed to underlie the CaMK-dependent regulation of sperm motility [46]. In *Chlamydomonas*, Ca^2+^ controls dynein-driven microtubule sliding by activating calmodulin and CaM kinase [53], [54]. Based on these findings, we propose a model where CRIS regulates protein transport into the axoneme during spermiogenesis through interaction with the transport machinery. In the absence of CRIS, flagellar proteins might be incorrectly assembled or lacking altogether.

## Discussion

Fertilization in mammals requires that sperm locate the egg. In the female reproductive tract, sperm are presumably guided by different mechanisms involving chemotaxis, thermotaxis, and rheotaxis [40], [55]–[57]. A precise control of sperm motility is a prerequisite for sperm to reach the site of fertilization and to locate the egg and, thereby, is instrumental for the success of fertilization. Here, we present a new regulator of sperm motility called CRIS.

CRIS exists predominantly in species with sperm that are propelled by the beat of the flagellum, but not in nematodes like *C.elegans* with sperm that do not carry a flagellum and crawl in an amoeboid fashion [58]. This suggests that CRIS is involved in maintaining flagellar function of sperm. Indeed, sperm from *Cris*-deficient mice display aberrant flagellar bending: their flagellar waveform is highly asymmetrical in the anti-hook conformation. As a result, sperm from mutant males show a substantially reduced swimming velocity along a straight path under both capacitating and non-capacitating conditions. Thus, progressive swimming is strongly reduced. Could this phenotype account for the fertility defect? Successful fertilization requires multiple factors; however, a prerequisite is that sperm reach the site of fertilization. A similar sperm phenotype as in *Cris*-deficient mice is observed in t haplotype mice [59]. Mouse t haplotypes are naturally occurring variants of chromosome 17 [60]. Sperm from t haplotype mice display a number of defects resulting in infertility. First, progressive swimming is impaired: curvilinear swimming velocity is only 50% and progressive swimming only 20% of that of wild-type sperm. Second, sperm display the “curlicue” phenotype - an augmented effect of Ca^2+^ on the axoneme, resulting in an anti-hook bending of the midpiece [59], [61]. Third, t haplotype sperm are not able to fuse with the egg, even when artificially transferred to the site of fertilization [62], [63]. Transport studies of these sperm in the female reproductive tract revealed that hardly any sperm are found in the oviduct after mating, indicating that few if any sperm reach the site of fertilization [64]. It was suggested that low progression of these sperm is the primary cause of sterility [64]. Although both the motility phenotypes and the abnormal flagellar bending of sperm from *Cris*-deficient and t haplotype mice are similar, *Cris*-deficient sperm can fertilize the egg during IVF. Thus, the t haplotype is more complex and likely involves several genes that contribute to sterility. *Cris* is located on chromosome 2, ruling out a direct effect of the t haplotype variants on *Cris* gene-function; however, CRIS might regulate proteins, whose function is also affected in t haplotype mice.

Recently, the male contribution to the fertility of the FL1 mouse line was investigated [65]. This mouse line was bred for the phenotype “high fertility” over 158 generations. During this period, the litter size per offspring increased from 10.4 to 17.1 [65]. Diallelic breeding demonstrated that about two third of the improved fertility phenotype is contributed by the male. Overall, fewer FL1 sperm were motile and progressive compared to wild-type mice; however, if motile, FL1 sperm displayed improved progression [65]. Taking the results from t haplotype, *Cris*-deficient, and FL1 mice together, sperm progression through the female genital tract is one of the major determinants of successful fertilization.

In sperm from both *Cris*-deficient and t haplotype mice, the defect in flagellar bending reflects a change in the Ca^2+^ regulation of flagellar bending rather than altered Ca^2+^ levels. Ca^2+^ is an important determinant of flagellar bending in general [66]–[68]. In mammalian sperm, Ca^2+^ entry via the sperm-specific CatSper channel underlies hyperactivated motility [43], [44]. It has been proposed that distinct Ca^2+^-signaling pathways control flagellar bending of mouse sperm in either the anti- or pro-hook conformation [35]: Ca^2+^ entry through CatSper channels predominantly induces pro-hook bending, whereas Ca^2+^ release from internal stores favors anti-hook bending [35]. Downstream of Ca^2+^, flagellar bending is governed by Ca^2+^-binding proteins that indirectly regulate the axonemal motor protein dynein [53], [54]. Ca^2+^-binding proteins that might control sperm motility are CaM, enkurin, and calaxin [69]–[71]. Calaxin is highly conserved in metazoa and has been first identified in *Ciona intestinalis* [71]. Calaxin binds to outer dynein arms and directly suppresses microtubule sliding at high Ca^2+^ concentrations, which is essential for Ca^2+^-induced asymmetric bending [71]. Enkurin binds to ion channels and to CaM, creating a Ca^2+^-sensitive signaling platform that has been suggested to control sperm function [70]. Activation of CaMKIV by CaM has been proposed to regulate sperm motility in humans [46]. Interestingly, we identified CaMKIV as a putative interaction partner for CRIS during spermatogenesis (Table 2). We tested the role of CaMKIV in controlling flagellar bending by incubating tethered wild-type sperm with KN93, a CaMKIV blocker. We observed a decrease in the asymmetry index of flagellar bending compared to control sperm. This is opposite to sperm from *Cris*-deficient mice, which show a higher asymmetry index compared to wild-type sperm. Thus, if CRIS controls CaMKIV function and, thereby, flagellar bending, our results suggest that the effect is rather to repress than to promote CaMKIV function.

However, one has to consider that our data do not support expression of CRIS in mature sperm. Therefore, CRIS needs to fulfill its role during sperm development, presumably in a cAMP-dependent manner. The flagellum is formed at the round spermatid stage, which temporally overlaps with CRIS expression. During this stage, proteins are transported into nascent cilia or flagella by the intraflagellar transport machinery [48], [72]. Some of the flagellar proteins, e.g. dynein, are pre-assembled in the cytoplasm before being transported into the flagellum [73]. Our interaction studies provide evidence that CRIS interacts with proteins that control intraflagellar transport (e.g. IFT172, KIF2A). CRIS is a cytosolic protein in spermatids and could, therefore, regulate the pre-assembly of the Ca^2+^-sensing proteins before they are transported into the sperm flagellum.

Recently, the role of two proteins controlling IFT in mouse sperm has been described. RABL2 is a member of the RAS GTPase superfamily and interacts with the IFT machinery to transport a specific set of effector proteins to the sperm flagellum [74]. *Rabl2*-deficient male mice are infertile and their sperm are immotile. However, RABL2 effector proteins are still transported into the flagellum of *Rabl2*-deficient mice, but the transport is less effective, showing a 20–30% reduction of protein localization in the sperm flagellum.

KIF3A motor protein is responsible for IFT in ciliated cells, but its role in sperm has been ill defined. KIF3A knockout males are infertile due to severe morphological defects of the sperm head and flagellum, indicating that KIF3A is essential for sperm head and tail formation [75]. Interestingly, testis weight of KIF3A knockout males was reduced and KIF3A and its interaction partners show a similar developmental expression pattern as we describe for CRIS. In the same vein, RABL2 also shows a similar developmental expression pattern as described for KIF3A, but also for CRIS [74]. Thus, the few components that have been shown to be involved in sperm IFT are expressed at a similar time point as CRIS during development.

The importance of CRIS during sperm development is underlined by the fact that a population of *Cris*-deficient males is infertile due to a spermatogenic arrest. However, infertility in *Cris*-deficient males displays partial penetrance, thereby, creating two groups: one with normal testis weight and morphology, which are subfertile, and one with reduced testis weight and aberrant testis morphology, which are infertile. Partial penetrance of infertility has also been reported in other studies. Male mice lacking the α1b-adrenergic receptor can also be grouped according to their fertility defect: 27% of the males are infertile, whereas 73% are subfertile [76]. Partial penetrance of infertility can be attributed to the segregation of genetic modifiers on a hybrid genetic background. Similar results were observed for *Hspa4*-null males lacking the heat-shock protein 4 [77], for mice lacking the transition nuclear protein 1 (Tnp) [78], or the POU protein sperm-1 [79]. The *Cris*-deficient mouse line has been backcrossed into the C57Bl/6 background for 8–10 generations. Thus, the segregation of genetic modifiers on a hybrid genetic background, which could affect the penetrance of infertility, is minimized, but cannot be excluded and could account for the partial penetrance of infertility in *Cris*-deficient male mice.

The reproductive phenotype observed in *Cris*-deficient mice is remarkably similar to that observed in some infertile men suffering from oligoasthenospermia, which is characterized by a lower sperm count and severe motility defects, but normal sperm morphology. Large-scale sequencing approaches need to address whether mutations in the *Cris* gene underlie fertility defects in human patients. Mice as model system are characterized by a high fecundity. Even if only 59% of *Cris*-deficient males are fertile, the population can be maintained. In humans, however, a similar defect might result in more severe phenotype and determines the success of a couple to conceive a child.

## Material and Methods

### Ethics statement

All animal experiments were in accordance with the relevant national and international guidelines and regulations. Animal procedures were approved by the local authorities (LANUV NRW).

### Phylogenetic analysis

CRIS orthologs were identified by a protein blast (blastp) in NCBI and an ortholog search in Ensembl (release 65). All sequences were verified using a reciprocal BLAST. CRIS protein sequences were aligned with MAFFT 4.0. The NJ algorithm was used to construct a phylogenetic tree with ClustalX [80]. Bootstrapping was performed 1,000 times using ClustalX. The tree was visualized with Dendroscope. Bootstrap values are given as percentages. The scale bar shows the amino-acid substitution rate for the NJ (neighbor joining) tree.

### Modeling of the CNBD

We used a region encompassing amino-acid residues 202–333 of mCRIS for homology modeling. The structure modeling-server M4T (M4T: a comparative protein structure modeling server (http://manaslu.aecom.yu.edu/M4T/) [81] was used to investigate the relationship of the CNBD of CRIS with those of other cyclic nucleotide-regulated proteins. A model was built based on the CNBDs of a sea urchin HCN channel (SpIH, 2ptm) [10] and of EPAC2 (3cf6) [11]. Model quality was checked using the ProQ quality prediction server [82]. The LGscore and MaxSub value of the model were 2.5 and 0.4, respectively, indicating good to very good model quality. Figures were generated using Maestro (Suite 2011: Maestro, version 9.2, Schrödinger, LLC).

### Cloning

The CNBD protein sequence from the hyperpolarization-activated and cyclic nucleotide-gated ion channel 4 (HCN4) was used to search the database. Regions of a novel sequence (ensemble: 4921517L17Rik) were similar to the CNBD sequence. We used primers derived from this sequence (Table 3) for PCR after reverse transcription of RNA (RT–PCR) from mouse testis to obtain cDNA. For heterologous expression, a hemagglutinin (HA) tag was fused to the C terminus (mCRIS-HA) and cloned into a pcDNA3.1+ vector (Invitrogen). Sequence alignments were done using ClustalW2. The FRET cer-mCNBD-cit construct was generated by PCR (aa 202–353, Table 3) using mCRIS cDNA as template. Citrine was fused to the N terminus and cerulean tagged with a histidine tag (His_10_), to the C terminus of the CNBD. GFP variants were amplified with standard primers from pEYFP and pECFP (Clontech). The mutated cer-mCNBD-R288Q-cit construct was generated using a QuikExchange protocol (Table 3, Stratagene).

**Table 3:** Primer sequences.

5′→3′ Primer sequence	Usage
CGGGGATCCACCATGAACCGATCCGC	5′ primer fragment 1 cDNA mCRIS
AGCAATCTAGAGCCTGAAG	3′ primer fragment 1 cDNA mCRIS
CTTCAGGCTCTAGATTGCT	5′ primer fragment 2 cDNA mCRIS
GGTGTTGACCATGGAACAT	3′ primer fragment 2 cDNA mCRIS
ATGTTCCATGGTCAACACC	5′ primer fragment 3 cDNA mCRIS
CTTTACTCGAGCTAAATAAGGACCCC	3′ primer fragment 3 cDNA mCRIS
GAACAGAATACTTTGAGCTGGC	5′ primer probe 1 Southern blot analysis ES cells
TAAATAAAGAAGCACTGGATTGC	3′ primer probe 1 Southern blot analysis ES cells
CGATAGAAGGCGATGCGCTG	5′ primer Neo probe Southern blot analysis ES cells
GCCATTGAACAAGATGGATTGC	3′ primer Neo probe Southern blot analysis ES cells
GCCTTCTTGACGAGTTCTTCTGAGG	5′ primer PCR analysis ES cells
CTTATGAGCTCCCAACTATGGCTGC	3′ primer PCR analysis ES cells
CCAAGACCTCTGCAATATTCTTC	5′ primer genotyping *Cris*-deficient mice
ACACTTGAAAAGTATGTGAACTAG	3′ primer wild-type fragment genotyping *Cris*-deficient mice
GCCTCTCCACCCAAGCGG	3′ primer recombinant fragment genotyping *Cris*-deficient mice
CCGCTCGAGATGGTGAGCAAGGGCGAG	5′ primer cerulean cer-mCNBD-cit
GCAGGGCCCTAGTGATGGTGATGGTGATGATGGTGATGATGC	3′ primer cerulean cer-mCNBD-cit
CCCAAGCTTCCACCATGGTGAGCAAGGGCGAGGAG	5′ primer citrine cer-mCNBD-cit
CGGGATCCCTTGTACAGCTCGTCCATGCCGAGAGTGATCCC	3′ primer citrine cer-mCNBD-cit
CGGGATCCCAGGCTCTAGATTGCTACC	5′ primer mCNBD cer-mCNBD-cit
CCGCTCGAGCCTCTCTATCCTCCCCAAG	3′ primer mCNBD cer-mCNBD-cit
GGGCCTTCTAAGCACCACAGTACAGAGTGCCACGGT	5′ primer R288Q cer-mCNBD-cit
ACCGTGGCACTCTGTACTGTGGTGCTTAGAAGGCCC	3′ primer R288Q cer-mCNBD-cit

The annotated rat ortholog (NM_001177681) is predicted to encode a ribosomal subunit, however, we could not amplify this sequence from rat testis cDNA.

### FRET measurements

Live-cell imaging was performed using CHO cells on the Olympus CellR system with an IX81 microscope, the MT20 illumination system, and the XM10 CCD camera. CHO cells expressing the FRET sensors were perfused with buffer (140 mM NaCl, 5.4 mM KCl, 1 mM MgCl_2_, 1.8 mM CaCl_2_, 5 mM HEPES) with or without 40 µM NKH477, 100 µM IBMX (Tocris), and 3 mM 8-Br-cAMP/cGMP (BioLog) 1 min after starting the recording. Cells were excited at 430/25 nm. Fluorescence was detected through 470/24 nm and 535/30 nm bandpass filters. Data was analyzed using the CellR software and Origin (Microcal), expressed as ratio of cerulean to citrine signal, and normalized to the values before perfusion.

### Antibody generation

A peptide comprising amino acids _142_VHKKHPDFSFWDKKKQGR_159_ from mouse CRIS was synthesized and coupled to BSA and OVA (PSL). Rats were immunized subcutaneously and intraperitoneally with a mixture of 50 µg peptide-OVA, 5 nmol CPG oligonucleotide (Tib Molbiol), 500 µl PBS, and 500 µl *incomplete Freund's* adjuvant. A boost without adjuvant was given six weeks after the primary injection. Fusion was performed using standard procedures. Supernatants were tested in a differential ELISA with the CRIS peptide coupled to BSA and irrelevant peptides coupled to the same carrier. Monoclonal antibodies that reacted specifically with CRIS were further analyzed in Western blot. Tissue culture supernatant of 4E4 and 4B2, both of rat IgG1 subclass, were used in this study. A polyclonal antibody (1880-1) directed against the same peptide was generated in rabbit and purified using a peptide column.

### Generation of CRIS^−/−^ mice

The targeting vector was constructed in a modified pBluescript vector (Stratagene) using a 2.7 kb genomic fragment as short arm of homology (exon 4+first 95 bp of exon 5) and a 6.6 kb genomic fragment as long arm of homology (last 134 bp of exon 7+exon 8). In between both arms, a loxP flanked neomycin resistance-gene was inserted, which replaced the 3′ end of exon 5, exon 6 and the 5′ end of exon 7. For negative selection, a diphtheria toxin A (DTA) cassette was inserted at the 3′ end of the long arm. The targeting vector was linearized with SwaI before electroporation into V6.5 embryonic stem cells [83]. Positive clones were identified by Southern blot analysis using probe 1 and a neo probe, and by PCR (Table 3). Eight-cell embryo injection was performed for two independent clones. Male chimeras were crossed with C57BL/6J females to obtain heterozygous mice. Mice were genotyped by PCR (Table 3). All mice were backcrossed for 10 generations to a C57BL/6J background. Mice used in this study were 2–4 months of age.

All animal experiments were in accordance with the relevant guidelines and regulations.

### Northern blot analysis

Northern blot analyses were performed using a mouse multi-tissue (Clontech) and a mouse reproductive-tissue Northern blot (Zyagen) labeled with a *Cris*-specific probe (bp 9–325). Northern blots were prehybridized at 68°C for at least 1 h using ExpressHyb solution (Clontech) and PerfectHyb Plus Hybridization buffer (Sigma), respectively. To detect mCRIS mRNA, a ^32^P labelled DNA probe (bp 9–325; TAKARA Labeling Kit) was used at 1.5–2×10^6^ cpm/ml in prehybridization solutions at 68°C overnight. After washing (three times in 0.05% SDS in 2×SSC, once in 0.1% SDS in 0.1×SSC, each 20 min at 65°C), signals were detected using Kodak® BioMax™ XAR films (Sigma).

### *In situ* hybridization

Digoxigenin (DIG)-labeled sense and antisense probes were synthesized by *in vitro* transcription using DIG RNA labeling mixes (Roche). The same cDNA region as for the Northern blot probe was used. Frozen testis sections (18 µm) were fixed for 10 min with 4% paraformaldehyde in PBS. After prehybridization in 50% formamide, 10% dextran sulfate, 100 µg/ml herring sperm DNA in 2×SSC, sections were hybridized with 25–100 ng probe in the same buffer at 42°C overnight. The sections were washed for 30 min at 42°C in 50% formamide/2×SSC and in 50% formamide/0.2×SSC. For detection, sections were equilibrated in buffer 1 (100 mM Tris/HCl, pH 7.5, 150 mM NaCl), blocked with blocking buffer (0.5% blocking reagent (Roche) in buffer 1) for 30 min at room temperature, and incubated with 0.15 U/ml sheep alkaline-phosphatase (AP)-conjugated anti-DIG Fab-fragments (Roche) in blocking buffer for 1 h at room temperature. After washing twice with buffer 1 and equilibration with buffer 3 (100 mM Tris/HCl, pH 9.5, 100 mM NaCl, 150 mM MgCl_2_), NBT/BCIP (nitroblue tetrazolium chloride/5-bromo-4-chloro-3-indolyl-phosphate, toluidine salt, Roche) was added to detect bound AP.

### Immunohistochemistry/immunocytochemistry

Testes were either directly embedded in TissueTec (Sakura Finetek), frozen, and sections (18 µm) were fixed for 10 min with 4% paraformaldehyde/PBS or Histochoice (Amresco) or were fixed overnight, cryo-protected in 10 and 30% sucrose, and afterwards embedded in TissueTec. Sperm were immobilized on microscope slides with or without Mitotracker (1 ng/µl; Invitrogen) and fixed for 10 min. To block unspecific binding sites, frozen sections and sperm were incubated for 1 h with blocking buffer (0.5% Triton-X 100 and 5% ChemiBLOCKER (Millipore) in 0.1 M phosphate buffer, pH 7.4). Primary antibodies (Table 4) were diluted in blocking buffer and incubated overnight. Fluorescent secondary antibodies were diluted in blocking buffer containing 0.5 µg/µl DAPI (Invitrogen) and pictures were taken on a confocal microscope (Olympus FV1000). Periodic acid Schiff's staining was performed following the manufacturer's instruction (Sigma). All antibodies are described in Table 4.

**Table 4:** Antibodies and their dilutions.

Primary Antibody	Produced by	Dilution in IB	Dilution in IF
4E4/4B2, rat monoclonal	E. Kremmer	undiluted	undiluted
1880-1, rabbit purified antiserum	Peptide Specialty Laboratories	1∶2000	1∶2000
calnexin, rabbit polyclonal C4731	Sigma	1∶50000	-
β-Actin [AC15], mouse monoclonal ab6276	Abcam	1∶50000	-
β-tubulin, mouse monoclonal T4026	Sigma	1∶1000	-
Phospho-PKA substrate, mouse monoclonal 9624	Cell Signaling Technology	1∶1000	-
Phosphotyrosine, clone 4G10, purified mouse monoclonal 05–321	Millipore	1∶1000	-
MIWI, rabbit polyclonal	NEB		1∶200
IFT172, rabbit polyclonal	Santa Cruz	1∶1000	1∶100
KIF2A, rabbit polyclonal	Millipore	1∶1000	1∶100
ABCF2, rabbit polyclonal	Antibodies online	1∶5000	1∶100

Secondary Antibody (conjugate)	Produced by	Dilution in IB	Dilution in IF
Goat-anti-rabbit (HRP)	Jackson ImmunoResearch	1∶5000	-
Goat-anti-rat (HRP)	Jackson ImmunoResearch	1∶5000	-
Goat-anti-mouse (HRP)	Jackson ImmunoResearch	1∶5000	-
Donkey-anti-rat (Cy3)	Jackson ImmunoResearch	-	1∶400
goat-anti-rat (Cy3)	Jackson ImmunoResearch	-	1∶500
goat-anti-rabbit (alexa488)	Jackson ImmunoResearch	-	1∶500

IB, immunoblotting; IF, immunofluorescence.

### Immunoblot analysis and immunoprecipitations

For heterologous expression, HEK293 or CHO cells were transfected using Lipofectamine 2000 (Invitrogen).

Lysates were isolated by homogenizing the tissue and cells in lysis buffer (10 mM Tris/HCl, pH 7.6, 140 mM NaCl, 1 mM EDTA, 1% Triton-X 100, 1∶500 mPIC protease inhibitor cocktail) with a tissue homogenizer. After three freeze-thaw cycles, samples were incubated 30 min on ice and then centrifuged at 10,000×g for 5 min at 4°C.

Soluble proteins were isolated by homogenizing the tissue and cells in buffer A (20 mM HEPES, 20 mM NaCl, 1 mM EDTA, 0.1 mM EGTA, pH 7.4, 1∶500 mPIC) with a tissue homogenizer. After three freeze-thaw cycles, samples were incubated 10 min on ice and then centrifuged at 20,000×g for 15 min at 4°C.

All samples were heated for 5 min at 95°C prior to separation on SDS-PAGE. For Western blot analysis, proteins were transferred onto PVDF membranes, probed with antibodies and analyzed using a chemiluminescence detection-system. All antibodies are described in Table 4. For reprobing, membranes were incubated in stripping buffer (62.5 mM Tris/HCl pH 6.7, 100 mM β-mercaptoethanol, 2% SDS) for 30 min at 65°C, washed with PBS, and reprobed.

For (co-)immunoprecipitations, 500 µg protein (total lysates) was incubated with 0.1 ml of the antibody column (monoclonal antibody 4B2 or 4E4 covalently coupled to Protein G) overnight at 4°C, washed four times with lysis buffer, and eluted in 2× SDS-PAGE sample buffer.

### Identification of CRIS by mass spectrometry

Before mass spectrometry, proteins were separated on a SDS-PAGE and gel pieces were digested with trypsin. LC-MS/MS was performed on a Micromass CapLC liquid chromatography system and a quadrupole orthogonal acceleration time-of-flight mass spectrometer Q-TOF Ultima (Micromass) equipped with a Z-spray nanoelectrospray source. Peptide separation was performed on a capillary column (PepMap C18, 3 µm, 100 Å, 150 mm×75 µm i.d., Dionex). Data was acquired in a data-dependent mode using one MS scan followed by MS/MS scans of the most abundant peak. The MS survey range was m/z 350–1500 and the MS/MS range was m/z 100–2000. The processed MS/MS spectra (MassLynx version 4.0 software) and the MASCOT server version 1.9 (Matrix Science Ltd.) were used to search an in-house database, which contained the CRIS sequences. The mass tolerance of precursor and sequence ions was set to 100 ppm and 0.2 Da, respectively. The search includes variable modifications of cysteins with acrylamide and methionine oxidation. CRIS was accepted as identified if at least two tryptic peptide scores indicate identity or extensive homology.

### Identification of potential interaction partners of CRIS

LC-MS/MS analyses of gel-separated proteins were performed on a LTQ-Orbitrap hybrid mass spectrometer (Thermo Fisher) equipped with an Eksigent 2D nanoflow LC system (Axel Semrau GmbH). Tryptic peptides were separated on a capillary column (PepMap C18, 3 µm, 100 Å, 250 mm×75 µm i.d., Dionex, Idstein) at an eluent flow rate of 250 nl/min using a linear gradient of 2–50% acetonitrile in 0.1% formic acid (v/v). Mass spectra were acquired in a data-dependent mode with one MS survey scan (with a resolution 60,000) in the Orbitrap and MS/MS scans of the four most intense precursor ions in the LTQ. The MS survey range was m/z 350–1500. The dynamic exclusion time (for precursor ions) was set to 120 s and automatic gain control was set to 10^6^ and 20,000 for Orbitrap-MS and LTQ-MS/MS scans, respectively. The generated peak lists and the MASCOT server (version 2.2, Matrix Science) were used to search in-house against the SwissProt database (version 2010_10, contains 521,016 sequences, comprising 183,900,292 residues). A maximum of two missed cleavages was allowed and the mass tolerance of precursor and sequence ions was set to 10 ppm and 0.35 Da, respectively. Acrylamide modification of cysteine and methionine oxidation were considered as possible modifications. Scaffold (version 2.02, Proteome Software Inc.) was used to validate MS/MS based peptide and protein identifications and to generate non-redundant protein lists. Peptide identifications were accepted if they could be established at greater than 70% probability as specified by the Peptide Prophet algorithm. Protein identifications were accepted if they could be established at greater than 99% probability and contained at least two identified peptides. Based on decoy database searches, the false positive rate was estimated to be <1%.

### Analysis breeding performance

Wild-type, heterozygous, or homozygous males (with normal testis weight) were mated with wild-type females (C57Bl/6) for four weeks. Only homozygous males with normal testis weight were used for the analysis of breeding performance.

### Isolation of sperm and male germ cells

Sperm were isolated by incision of the cauda epididymis in modified TYH medium containing 138 mM NaCl, 4.8 mM KCl, 2 mM CaCl_2_, 1.2 mM KH_2_PO_4_, 1 mM MgSO_4_, 5.6 mM glucose, 0.5 mM sodium pyruvate, 10 mM L-lactate, pH 7.4. For capacitation, the medium contained 3 mg/ml BSA and was supplemented with 25 mM NaHCO_3_. After 15 min swim out at 37°C and 5% CO_2,_ sperm were counted. All subsequent experiments were performed at room temperature, unless otherwise stated.

For isolation of germ cells, testes were decapsulated and incubated in 1 ml Hank's Balanced Salt Solution (HBSS) containing (20 mM HEPES, 137 mM NaCl, 5.4 mM KCl, 0.3 mM Na_2_HPO_4_, 0.4 mM KH_2_PO_4_, 1.2 mM MgSO_4_, 1.3 mM CaCl_2_, 6.6 mM sodium pyruvate, 0.05% lactate, 5.6 mM glucose, pH 7.2) containing 0.5 mg/ml Collagenase type IA (Sigma) for 30 min at 32°C. The dissociated interstitial cells were removed by two washing steps with HBSS. The seminiferous tubules were then incubated in 1 ml HBSS containing 0.5 mg/ml Trypsin type XIII (Sigma) and 1 µg/ml DNaseI (Applichem) for 10 min at 32°C. Cell aggregates were sheared gently with a Pasteur pipette. The dispersed seminiferous cells were washed twice by centrifuging at 200×g for 5 min at room temperature. The final cell pellet was resuspended in HBSS and filtered through a Nylon mesh (40 µm mesh).

### *In vitro* fertilization

*In vitro* fertilization (IVF) was performed as described previously [84] and in cooperation with MfD Diagnostics, Wendelsheim, Germany.

### DNA content analysis

DNA content analysis using flow cytometry (FACS) was performed as described previously [85].

### Sperm motility analysis

Sperm motility was studied in shallow observation chambers (depth 150 µm). Cells were either freely swimming or tethered to the glass surface at a lower BSA concentration (0.3 mg/ml), which resulted in a large fraction of cells that gently adhered to the glass surface. For analysis, we selected those cells attached by the head only and displayed a freely beating flagellum.

Sperm motility was recorded under an inverted microscope (IX71; Olympus) equipped with a dark-field condenser and a 10× objective (UPLSAPO; NA 0.4). The temperature of the microscope was adjusted to 37°C using an incubator (Life Imaging Services). To obtain sharp images of moving sperm, stroboscopic illumination was achieved using a white LED (K2 star; Luxeon) and a custom-made housing and pulse generator (freely swimming: 1 ms, tethered: 2 ms). Images were collected using an EMCCD camera (DU-897D; Andor) for all data shown with the exception of data shown in Figure 5, where a CMOS camera (Dimax; PCO) was used instead. Cells where manually tracked during acquisition using a motorized stage (SCAN IM; Märzhäuser), and the position of the stage was recorded for each frame. The set-up was synchronized using a custom-made acquisition program written in LabVIEW and data acquisition hardware PCI-6040E (National Instruments).

Quantification of the flagellar beat was performed using custom-made programs written in MATLAB (Mathworks). The program identified the best threshold for binarization of the image by iteratively reducing the threshold until the expected cell area in the image was achieved. This was followed by a skeleton operation to identify the flagellum. The flagellar beat parameters were averaged during a time window of 1 s around each frame (except for frames at the beginning, the end of the movie, or flanking the UV flash, where the time window was reduced to ∼300 ms). The flagellar asymmetry index was defined as the angle between the line going through the middle of the flagellum and the sperm head and the axis of symmetry of the cell. For alignment of the flagellar-beat envelopes, we used custom-made programs written in LabVIEW. Using defined thresholds, the image was binarized. From a user-defined region-of-interest centered at the cell head, the program determined the location of the head on subsequent frames using a registering procedure. The neck of the cell was identified by applying a mask with the shape of an annulus centered into the sperm head. The annulus had an internal diameter of 16 µm to cover the sperm head, and a 4 µm longer external diameter, enough to resolve the first pixels of the neck. All frames were then rotated and superimposed with a rotation angle equal to the azimuth of the neck region on a reference system centered at the sperm head.

### 2-Diazo/Fluo-8 measurements

Sperm were loaded with 2-Diazo-AM (20 µM; Molecular Probes) for 40 min at 37°C. After incubation, cells were centrifugated (600×g, 8 min), resuspended in TYH buffer containing a lower BSA concentration (0.3 mg/ml), and allowed to tether onto the chamber wall. Photolysis of 2-Diazo was achieved by 100–200 ms UV flashes from a solid-state light source (Spectra×light engine, Lumencor) and a band-pass filter (H350/50; AHF). Images were collected at 95 frames per second.

For Ca^2+^ recordings, sperm were loaded with Fluo8-AM (5 µM; AAT Bioquest) together with Diazo-2. Excitation of fluorescence was achieved by stroboscopic illumination pulses (1 ms) generated with the cyan light-source of the light-engine Spectra X. Excitation light was filtered through a 475/28 band-pass filter (Bright Line HC, Semrock) and emission was long-pass filtered (510 ALP, Omega optical). Images were collected at 12 frames per second.

### Cryo-electron microscopy

Sperm were cryo-fixed by high-pressure freezing in 20% dextran (Dextran 40, Mr 40 kDa, Roth) [86], [87]. Frozen samples were freeze-substituted with 0.2% uranyl acetate in acetone [88]. Finally, samples were infiltrated with Lowicryl (EMS) and polymerized under UV light for several days. Testis sections (50 nm) were generated using an ultra-microtome (UC6, Leica Microsystems), collected on Quanifoil-Cu-grids (EMS), and stained with 2% uranyl acetate and 0.03% lead citrate. Images were taken with the JEM-2200FS transmission electron-microscope (TEM, JEOL) operating at 200 kV. The tomogram was acquired using the Titan Krios TEM (FEI Company) operating at 300 kV. The tomogram was reconstructed with eTomo [89].

### Calibration of [Ca^2+^]_i_

Sperm were loaded with 5 µM Cal-520-AM (ATT Bioquest), 0.05% pluronic (Invitrogen) in TYH buffer for 45 min at 37°C. After loading, sperm were washed three times with TYH buffer without lactate before starting the measurement. Calibration of [Ca^2+^]_i_ was performed using a null point method in a rapid-mixing device in the stopped-flow mode (SFM400; Bio-Logic) at 37°C. Sperm were mixed with defined Ca^2+^ solutions containing ionomycin (1 µM final). The [Ca^2+^]_i_ equaled the Ca^2+^ concentration, at which no change in fluorescence was observed.

### Statistics

All values are mean ± s.d., unless otherwise stated. Statistical comparisons were carried out with Student's t test. Statistically significance was accepted at the p<0.05 level. The 95% confident interval was calculated as 1.96×s.e.m.

## Supporting Information

Movie S1 Tomogram of a cross-section through the midpiece of a mutant sperm.
(AVI)

Movie S2 Freely swimming sperm (wild-type and *Cris*-deficient) under non-capacitating conditions. The recording was performed using an epifluorescent microscope (IX71; Olympus) equipped with a 10× objective (UPLSAPO; NA 0.4). Frames were acquired at 60 fps using a back-illuminated EMCCD camera (DU-897D; Andor Technologies). The movie is shown in real time.
(AVI)

Movie S3 Freely swimming sperm (wild-type and *Cris*-deficient) under capacitating conditions. The recording was performed as in Movie S2.
(AVI)

Movie S4 Diazo-2 loaded wild-type sperm before and after UV flash. The cell was gently tethered to the glass surface by lowering the BSA concentration (0.3 mg/ml). The recording was performed using an epifluorescent microscope (IX71; Olympus) equipped with a 10× objective (UPLSAPO; NA 0.4) and additional 1.6× lenses. Frames were acquired at 95 fps using a back-illuminated EMCCD camera (DU-897D; Andor Technologies).
(AVI)

Movie S5 Diazo-2 loaded *Cris*-deficient sperm before and after UV flash. The recording was performed as in Movie S4.
(AVI)
